# Linking canonical microcircuits and neuronal activity: Dynamic causal modelling of laminar recordings

**DOI:** 10.1016/j.neuroimage.2016.11.041

**Published:** 2017-02-01

**Authors:** D.A. Pinotsis, J.P. Geerts, L. Pinto, T.H.B. FitzGerald, V. Litvak, R. Auksztulewicz, K.J. Friston

**Affiliations:** a The Picower Institute for Learning & Memory and Department of Brain and Cognitive Sciences, Massachusetts Institute of Technology, Cambridge, MA 02139, United States; b The Wellcome Trust Centre for Neuroimaging, University College London, Queen Square, London WC1N 3BG, UK; c Helen Wills Neuroscience Institute, University of California, Berkeley, CA 94720, United States; d MPS – UCL Centre for Computational Psychiatry and Ageing Research, Russell Square House, London, WC1B 5EH, UK; e Oxford Centre for Human Brain Activity, Department of Psychiatry, University of Oxford, Oxford OX3 7JX, UK

## Abstract

Neural models describe brain activity at different scales, ranging from single cells to whole brain networks. Here, we attempt to reconcile models operating at the microscopic (compartmental) and mesoscopic (neural mass) scales to analyse data from microelectrode recordings of intralaminar neural activity. Although these two classes of models operate at different scales, it is relatively straightforward to create neural mass models of ensemble activity that are equipped with priors obtained after fitting data generated by detailed microscopic models. This provides generative (forward) models of measured neuronal responses that retain construct validity in relation to compartmental models. We illustrate our approach using cross spectral responses obtained from V1 during a visual perception paradigm that involved optogenetic manipulation of the basal forebrain. We find that the resulting neural mass model can distinguish between activity in distinct cortical layers – both with and without optogenetic activation – and that cholinergic input appears to enhance (disinhibit) superficial layer activity relative to deep layers. This is particularly interesting from the perspective of predictive coding, where neuromodulators are thought to boost prediction errors that ascend the cortical hierarchy.

## Highlights

• Biophysical mean field model fitted to data from different cortical layers.
• Neural mass model combined with a laminar-specific forward model.
• Fitted V1 data with and without optogenetic activation of the basal forebrain.
• Test of Predictive Coding based on the role of neuromodulation.
• Increase in prediction error activity due to cholinergic effects.

## 1 Introduction

Multi-electrode shanks and multi-unit probes provide a unique window on the functional microarchitecture of cortical and subcortical structures, like V1, temporal cortex, the hippocampus or the cerebellum, see e.g. (Olsson et al., 2005, Obien et al., 2014, Kelly et al., 2007, Ulbert et al., 2001). These recording techniques have found a wide range of applications, including brain-machine interfacing (Hiremath et al., 2015) and seizure localization (Halgren et al., 2015). They allow for simultaneous recordings from different layers within a single brain region and offer insights into the functional architecture, physiology and anatomy of cortical microcircuitry.

Laminar array recordings can be obtained using thin probes with multiple contacts that penetrate (almost) vertically the cortical surface. These recordings can be used to reconstruct synaptic activity and dendritic currents flowing between different layers. This reconstruction entails an (ill posed) inverse problem of mapping responses to laminar-specific neuronal sources. This mapping has been addressed using methods like Current Source Density (Freeman and Nicholson, 1975, Koo et al., 2015, Mitzdorf and Singer, 1977, Sakamoto et al., 2015) and more recently Laminar Population Analysis (Einevoll et al., 2007, Ness et al., 2015).

Here, we suggest an alternative approach to estimating layer-specific activity using Variational Bayesian deconvolution. We first obtain simulated responses from a compartmental model that has been previously shown to faithfully represent the cortical microarchitecture – and has been used to model MEG responses during a tactile stimulation paradigm (Bush and Sejnowski, 1993, Jones et al., 2007). We then use these simulated data to optimise the mean-field (lumped) parameters of a homologous neural mass model. The resulting parameters provide prior constraints on neural mass models that can be used for subsequent dynamic causal modelling of empirical responses. This approach ensures the neural mass model has construct validity, in relation to more detailed (compartmental) models of cortical microcircuitry.

The resulting neural mass model can be combined with an observation model that allows one to simultaneously fit predicted time series from different subpopulations within the same neural circuit. This contrasts with the current use of mean field models to generate (weighted) mixtures of responses in different populations, thereby providing a single time series for each cortical or subcortical source. The implicit mixing is appropriate for non-invasive electromagnetic recordings that cannot resolve the cortical depth of sources; however, for laminar data one needs to equip the observation model with spatial parameters that associate each population with a particular cortical layer. This leads to the natural question: do the neural masses that model superficial and deep pyramidal populations actually occupy supragranular and infragranular positions in the cortex? Hitherto, in the dynamic causal modelling literature, the designation of a population as superficial (or deep) is based purely on their characteristic time constants and connectivity, without any explicit reference to their spatial deployment. In this paper, we ask whether functional attributions like superficial and deep are justified, when one can actually measure neuronal responses at different cortical depths.

Our approach to this question relies upon Bayesian model comparison and assumes that a Bayes optimal explanation (model) of data exists for some prior distribution of mean field parameters. To ensure the prior constraints properly accommodate spatiotemporal dynamics within the cortical microcircuit and its neuronal compartments (e.g. delays due to spread of current throughout the dendritic arbours), the priors in this work were obtained by fitting a neural mass model to data generated by a validated compartmental model. In other words, we use the mean field homologue and its compartmental variant to find the prior distribution that renders both models functionally equivalent: i.e., find priors that produce the same responses. This enables us to model laminar responses using a relatively small number of parameters that can be estimated more efficiently, using the mean field homologue of the compartmental model. The empirical data used to illustrate this approach were recorded during a visual perception paradigm – with optogenetic manipulation – and were analysed here by inverting cross spectral density data features using DCM (Friston et al., 2007, Pinotsis et al., 2013, Pinotsis et al., 2012a).

In summary, the key innovation described in this paper is to equip standard neural mass models with laminar specific forward models that enable the fitting of laminar recordings. To lend the neural mass model construct validity – in relation to more detailed compartmental models that accommodate neuronal interactions between layers – we optimised the (prior) parameters of a standard neural mass model to reproduce the behaviour of more detailed, compartmental models. Effectively, we are repurposing established neural mass models to explain the laminar specific recordings. The empirical analyses based upon the ensuing model, although purely illustrative, establish a degree of face and construct validity for this approach.

In what follows, we first provide a brief review of compartmental modelling of laminar specific and non-invasive electromagnetic responses. We then consider mean field approximations to compartmental models; with a special focus on homogenous and symmetric coupling among cortical mini columns. These mean field approximations allow us to fit neural mass models of the sort used in dynamic causal modelling to simulated data generated by detailed compartmental models. The subsequent sections of this paper consider two issues: first, how to construct a neural mass model that inherits biological plausibility from compartmental models. This issue is addressed by fitting a standard neural mass model to data generated by compartmental models – and using the resulting posterior parameter estimates as priors for subsequent neural mass modelling of empirical data. The second issue is how to establish the validity of the resulting neural mass model. Here, we provide some provisional analyses of empirical data looking at its ability to correctly identify laminar-specific neuronal activity – and to detect the cholinergic neuromodulation of superficial pyramidal cells.

## 2 Materials and methods

### 2.1 Compartmental models and mean field approximations

We first briefly review an established compartmental neural model (Bush and Sejnowski, 1993). These authors show how a detailed multi-compartmental model can be reduced to a simpler model with fewer compartments. This model was later extended to a network model of a cortical column in a key paper by (Jones et al., 2007). The resulting network model provides detailed descriptions of intracellular (longitudinal) currents within the long apical dendrites of synchronized cortical pyramidal cells, see e.g. (Bazhenov et al., 2002, Dayan and Abbott, 2001, Einevoll, 2014, Krupa et al., 2008, Lindén et al., 2010, Ramirez-Villegas et al., 2015, Roth and Häusser, 2001, Santaniello et al., 2015). In these compartmental models, neuronal populations are organised spatially into networks of mini-columns: each mini-column consists of principal neurons (PNs) whose somata are placed in supragranular and infragranular layers. The resulting pairs of cells are connected with each other and also with principal cells in neighbouring mini-columns – and receive inhibitory input from interneurons that are shared between mini-columns. In summary, the model described in (Jones et al., 2007) embodies the laminar structure of a cortical column and can characterize the cellular and circuit level processes that are measured with multielectrode arrays, MEG or electrocorticography. It also provides characterizations of neuronal morphology and how neurons are grouped together to form spatially extended networks of mini-columns with well-behaved intrinsic (inter-and intra-laminar) connectivity.

The network model we consider here was originally used to explain somatosensory evoked responses measured with MEG during a tactile stimulation paradigm (Jones et al., 2007). When challenged with the appropriate sequence of exogenous input, the model accurately reproduces the S1 evoked response to a tap on the hand. Furthermore, the compartmentalisation of the PNs allowed the authors to make accurate predictions about the origin of each peak. For instance, it led to the prediction that the evoked response was generated by a sequence of feedforward (FF) input from the lemniscal thalamus to the granular layer, followed by feedback (FB) drive from higher order cortex and a late thalamic input to L4. Importantly, the model accurately describes the intracellular currents that give rise to signal polarity. In later studies, the model was extended to 100 pyramidal neurons (PNs) per cortical layer, and has been used to investigate the emergence of beta and gamma rhythms (Jones et al., 2009, Lee and Jones, 2013).

The current dipole approach used in (Jones et al., 2007) has been shown to be a good approximation for analysing MEG or EEG data, see e.g. (Murakami and Okada, 2006) although less so for local field potentials (LFPs), see (Lindén et al., 2010). Below we use a mean field model to explain invasive data using DCM: see, (Brunel and Wang, 2003, Deco et al., 2008, Marreiros et al., 2015, Moran et al., 2015) and (Friston et al., 2015) for a review of mean field approaches to this sort of modelling. Mean field or neural mass approaches generally assume that dendritic and other microscopic effects do not dominate the LFPs. Furthermore, they can only model axonal and dendritic arbours as passive cables and cannot capture properties of active media, like back-propagation of action potentials. These are clearly simplifying assumptions; indeed, several studies have considered alternative models of LFP signals, e.g. (Holcman and Tsodyks, 2006, Mazzoni et al., 2008, Touboul and Destexhe, 2010).

In what follows, we construct a variant of the (Jones et al., 2007) model that assumes inhibitory and excitatory cells are uniformly distributed in space. Furthermore, we adopt symmetry constraints on horizontal connectivity (within each cortical layer) of the sort assumed in neural and mean field models (Pinotsis et al., 2015). This means that inhibition is homogeneously distributed over the cortical surface – as in neural field models – as opposed to inhibitory interneurons being shared between mini-columns – as in the original compartmental model. In other words, we assume that laminar specific populations are distributed uniformly within a local cortical manifold and render the network homogeneous. The assumption of homogenous coupling means that compartmental and mean field models can, in principle, explain the same responses. In turn, this implies that mean field models of the sort considered below provide sufficient descriptions of neural tissue activity, provided single units oscillate synchronously (Hämäläinen et al., 1993).

In terms of solving the inverse problem, this homogenous coupling assumption means that one can substitute a myriad of coupled equations for multiple compartments in multiple mini-columns by a few integrodifferential equations of the sort used in mean field theory – and consider an alternative (but equivalent) parameterization in terms of lumped model parameters. Using this mean field reduction one obtains a model which can be fitted to empirical data very efficiently. To test the assumption that a mean field model can reproduce ERPs obtained from a compartmental model with multiple mini columns, we compared the responses of both models to the same input to ensure that they are formally equivalent.

### 2.2 DCM for microelectrode data

Compartmental models of the sort discussed above yield precise descriptions of the anatomy, morphology and biophysical properties of the underlying neuronal populations. Crucially, they produce neuronal dynamics that embody detailed spatiotemporal processes like currents flowing along dendrites and axons. For example, we will see below that both the original model and its mean field or neural mass variant can explain the M25 and M135 peaks in evoked responses obtained during the somatosensory task analysed in (Jones et al., 2007). These response components are generated by currents flowing towards the superficial layers, i.e., toward the apical dendrites.

**Fig. 1:**
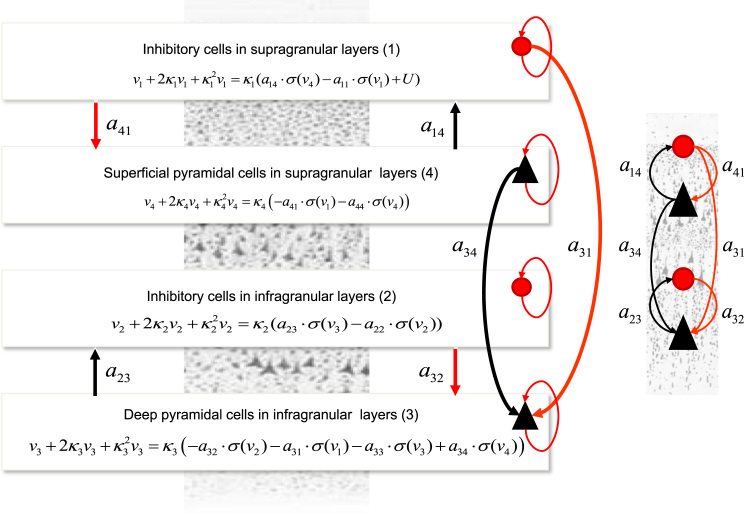
The Bush and Sejnowski (neural mass or mean field) model. This figure shows the evolution equations that specify a neural mass model of a single cortical microcircuit source. This model contains four populations occupying different cortical layers: the pyramidal cell population of the Jansen and Rit model is here split into two subpopulations allowing a separation of the sources of forward and backward connections in cortical hierarchies. Firing rates within each sub-population provide inputs to other populations and convolution of presynaptic activity produces postsynaptic depolarization. We consider separate time series of activity from superficial and deep populations as opposed to usual treatments that use weighted sums of activity from all subpopulations. Here red denotes inhibitory populations and connections, while black denotes excitatory cells and connections. Note that all recurrent or self connections are inhibitory.

In brief, neural mass models that are not equipped with distinct neuronal compartments cannot describe these detailed (dendritic or axonal) delays and back propagation. However, by fitting data generated by compartmental models using equivalent neural mass models – with the same number of populations and connectivity architecture – one can identify a prior distribution over model parameters that can reproduce the equivalent dynamics. This model is shown in Fig. 1 and comprises two pairs of coupled excitatory and inhibitory populations occupying the superficial and deep layers.

In the second part of our analysis, we consider the dynamic causal modelling of oscillatory responses using a neural mass model that inherits (prior) constraints from more detailed compartmental models. We analyse empirical recordings obtained from different cortical layers of the visual cortex using depth electrodes, during optogenetic stimulation of the basal forebrain. Our analysis focuses on the quantitative analysis of intrinsic (inter-and intra-laminar) connectivity and the effect of (putative) cholinergic stimulation. We restrict ourselves to describing and validating the neural mass model in this paper. This paper demonstrates that a neural mass model can be used to explain lamina-specific spectra observed during baseline and optogenetic manipulation. In a subsequent paper, we will use Bayesian model comparison to make inferences about the synaptic connections affected by cholinergic stimulation – and the resulting changes in cross spectral density.

In summary, we use the DCM in Fig. 1 to explain two different datasets: first, simulated data obtained from the Jones et al. model that has been shown to faithfully explain somatosensory evoked responses obtained with MEG during a tactile simulation paradigm. Second, LFP data obtained from V1 during optogenetic stimulation of the basal forebrain. The first dataset is used to establish that the DCM model can explain the same data as the model by Jones et al. The second dataset is used to test whether this DCM can also predict responses recorded from different cortical layers and the increase in prediction error activity due to cholinergic effects as suggested by the theory of predictive coding.

### 2.3 Local field potential data

We reanalysed data collected during a previously reported study; for more details see (Pinto et al., 2013). Briefly, 32 channel LFP data were acquired at a sampling rate of 581 Hz from Neuronexus A1×32-Poly2-5mm-50s-177 silicon probes implanted into V1 in 14 awake mice. Probe microelectrodes were arranged in two columns of 16. Spacing within columns was 50 μm, with a 25 μm between columns, giving a total length of 775 μm, sufficient to span the entire cortical thickness (approximately 800 μm in mice).

Data were acquired while the mice ran on a spherical treadmill and viewed a 7” LCD screen. The grating was static for 1 s, and then drifting for 4 s, giving a total trial length of 5 s. Gratings could be moving either sideways or upwards, and were presented at three contrast levels (20, 40, and 100%). 8 blocks of trials were presented. During four blocks, cholinergic neurons in the basal forebrain were optogenetically activated with five-second square laser pulses that accompanied the stimuli. LFP data were referenced to create 16 bipolar channels, and notch filtered at 60, 120 and 180 Hz to remove line noise. The cross spectral density was calculated separately for each trial, for the 4 second period between the onset of stimulus motion and stimulus offset, and averaged across trials.

### 2.4 Compartmental modelling

To construct a homogenous or *symmetric* compartmental model, we adapted NEURON code for the (Jones et al., 2007) model from the link below.^3^ Jones et al. (2007) modelled primary somatosensory cortex (S1) using reduced compartmental PNs, which allowed for an accurate description of dendritic currents (Bush and Sejnowski, 1993), and single compartment inhibitory interneurons (INs). These authors focused on simulating an evoked response to tactile stimulation of the hand, and computing the resulting current dipole (CD) signal – in order to characterise the local cortical dynamics that give rise to the MEG signal recorded over S1 during tactile stimulation. The model comprised 10 PNs in layers 2/3, 10 PNs in layer 5, and 3 INs in both layers. The synaptic architecture followed general tenets of cortical micro-circuitry (Douglas and Martin, 2004, Felleman and Van Essen, 1991), where FF connections target the granular layer and FB connections target agranular layers.

To determine whether the symmetric and original compartmental model generated the same responses, we increased the number of inhibitory units from three to 10 per layer, so that their number equalled the number of the principal cells within each mini-column. To ensure that relative differences in interneuron densities were accommodated, we multiplied the maximum conductance values of the corresponding connections by a factor of 0.3. Modelling of single neuron morphology and physiology followed (Bush and Sejnowski, 1993), using the same parameters as in (Jones et al., 2007). Following these earlier studies, input was provided by stochastic spike generators.

### 2.5 Single neuron morphology and physiology

Neocortical pyramidal neurons were modelled using reduced compartmental models with Hodgkin-Huxley type currents (Bush and Sejnowski, 1993, Jones et al., 2009, Jones et al., 2007, Lee and Jones, 2013)

(1) $$I_{m}=\sum_{l}g_{l}(V-E_{l})$$

where $g_{l}$ is the maximal conductance for channel *l,V* is the membrane potential and $E_{l}$ is the reversal potential. The superficial and deep PNs consisted of 8 or 9 compartments respectively, with compartment sizes and resistances as reported in Jones et al. (2007). PNs in both layers contained a fast sodium current (I_Na_), an adapting potassium current (I_M_), a delayed rectifier current (I_Kdr_) and a leak current (I_L_). In the L5 PNs a calcium-dependent potassium current (I_KCa_) and a calcium decay current (I_Ca_) with a decay time constant of 20 ms were present. The INs comprised a single compartment and contained only fast sodium (I_Na_) and potassium (I_Kdr_) currents. The reversal potentials and conductance for each current were identical to (Jones et al., 2007).

### 2.6 Local synaptic connections

Superficial and deep layers contained 10 PNs and 10 INs, and the location of synaptic inputs followed Jones et al. (2007). The synaptic dynamics of each connection were determined by their rise (*τ*_*1*_) and decay (*τ*_*2*_) time constants and reversal potential *E*. the synaptic current $I_{s}$ is given by the following equations:

(2) $$\begin{array}{c} I_{s}=g_{s}(V-E)\\ g_{s}=wf(e^{-t/\tau_{2}}-e^{-t/\tau_{1}}) \end{array}$$

where *f* is a normalising factor and $g_{s}$ is the synaptic conductance. Excitatory connections are mediated by AMPA (*τ*_*1*_=0.5 ms, *τ*_*2*_=5 ms, *E*=0 mV) and NMDA (*τ*_*1*_=1 ms, *τ*_*2*_=20 ms, *E*=0 mV) receptors. Inhibitory connections are mediated by GABA_A_ (*τ*_*1*_=0.5 ms, *τ*_*2*_=5 ms, *E*=−80 mV) and GABA_B_ (*τ*_*1*_=1, *τ*_*2*_=20, *E*=−80 mV) receptors. The weights of the synaptic connections w follow a Gaussian profile and an inverse Gaussian delay profile such that connections are stronger and faster for nearby cells, that is for connections between neurons at positions *i* and *j* the strengths and delay constants are given by

(3) $$\begin{array}{c} w=w_{\max}e^{-{\left|i-j\right|}^{2}/{C_{s}}^{2}}\\ d=d_{\min}e^{{\left(-{\left|i-j\right|}^{2}/{C_{d}}^{2}\right)}^{-1}} \end{array}$$

All local synaptic connection parameters and constants are listed in Table 1.

### 2.7 Exogenous inputs

The inputs innervating the network were separated into feedforward (FF) and feedback (FB), based on the canonical microcircuit (Douglas and Martin, 2004, Felleman and Van Essen, 1991). FF connections were modelled as granular layer (L4) input, originating in the thalamus (Jones et al., 2009, Jones et al., 2007). The FF drive comprised a connection to the basal and oblique dendrites of L2 PNs (as well as the L2 INs), and a delayed connection to the basal and oblique dendrites of L5 PNs and to the L5 INs. FB drive modelled input from higher-order cortex, and contacted the apical tufts of the PNs in both layers, as well as the L2 INs.

We consider inputs of two sorts: first, inputs generating evoked responses and second, ongoing subthreshold inputs that generate alpha activity. This involved expanding the model of (Jones et al., 2007) to produce oscillatory activity: following (Jones et al., 2009), 10 Hz thalamic (forward) inputs were obtained by simulating groups of 10 bursts (each consisting of 2 spikes separated by a 10 ms interval) with 100 ms intervals between the burst groups (Andersen and Andersson, 1968, Contreras and Steriade, 1995). Model output included current dipole sources that report the electrical activity of superficial and deep layers.

Input timings were chosen as reported in (Jones et al., 2009, Jones et al., 2007), and consistent with laminar recording data (Cauller and Kulics, 1991, Kandel and Buzsáki, 1997). These followed a Gaussian distribution across trials and consisted of an early FF drive around 25 ms (σ=2.5), followed by a FB input around 70 ms (σ=6) and a later wave of FF input (LFF) around 135 ms (σ=7). Each input comprised a single presynaptic spike, with suprathreshold synaptic weights as listed in Table 2. Note that contrary to Jones et al. (2007), the synaptic weights and delays of exogenous inputs were homogenously distributed over all cells. Noise or random fluctuations were modelled with a stochastic current between −0.3 and 0.3 nA to each compartment.

Earlier extensions of the (Jones et al., 2007) model have demonstrated rhythmogenesis and oscillations in the alpha, beta (Jones et al., 2009) and gamma (Lee and Jones, 2013) bands. We focused on reproducing alpha band activity with a symmetric model as follows. The model was driven with ongoing rhythmic FF drive, where each burst consisted of 2 spikes with an inter-spike interval of 10 ms, which is consistent with recording data (Hughes and Crunelli, 2005), and an inter-burst interval of (on average) 100 ms. The arrival time of each burst followed a Gaussian distribution with a standard deviation of 20 ms. In addition, FB with the same temporal statistics, but a 5 ms delay compared to the FF input, was added. The conductance was the same for FF and FB inputs: 0.4 picosiemens (pS) for input to PNs and 0.8 pS to INs. These parameters were chosen to ensure that all oscillations remained below the firing threshold.

### 2.8 Neural modelling and Bayesian Inversion

In addition to the symmetric compartmental model above, we constructed a neural mass variant of the (Jones et al., 2007) model for subsequent Dynamic Causal Modelling. Laminar-specific recordings call for a novel parameterisation of the observation model or lead field that changes with depth. In this setting, laminar LFP responses $y_{i}$ – of the sort measured with multi-electrode shanks – are generated by contributions from excitatory and inhibitory populations that occupy one or more cortical layers, see also (Pinotsis et al., 2012b, Pinotsis et al., 2014).

(4) $$y_{i}(t,\theta )=\underset{m}{\sum }L_{m}(\varphi )v_{m}(t),m=1,...,4{\;\overset{¨}{v}}_{m}=-2\kappa_{m}{\overset{}{v}}_{m}-\kappa_{m}^{2}v_{m}+\kappa_{m}f_{m}(v_{m},U,\theta ){\;f}_{m}(v_{m},U,\theta )=\{\begin{array}{cc} a_{14}\cdot \sigma (v_{4})-a_{11}\cdot \sigma (v_{1})+U & m=1\\ a_{23}\cdot \sigma (v_{3})-a_{22}\cdot \sigma (v_{2}) & m=2\\ -a_{32}\cdot \sigma (v_{2})-a_{31}\cdot \sigma (v_{1})-a_{33}\cdot \sigma (v_{3})+a_{34}\cdot \sigma (v_{4}) & m=3\\ -a_{41}\cdot \sigma (v_{1})-a_{44}\cdot \sigma (v_{4}) & m=4 \end{array}$$

Here, $L_{m}(\varphi )$ is a lead field describing the spatial sensitivity (tangential to the cortical penetration) of a microelectrode contact in layer *m*, (φ,θ) are lead field and neural mass parameters respectively, $v_{m}(t)$ is the depolarization of the population in layer *m,* and σ is a sigmoid operator transforming it into firing rate. $\kappa_{m}$ is the matrix of rate constants associated with postsynaptic processing and *U* stands for the inputs to local cortical circuit, see Table 3 for a list of biophysical parameters and their prior expectations.

For the neural mass models used below, we assumed that there was a one-to-one mapping between the recordings from an electrode in layer *i* and the depolarisation of the corresponding population. In other words, $L_{i}(\varphi )=\varphi_{j}:\forall i=j$ and zero otherwise. In other words, the indices in Eq. (4) play the same role; i≡m. This allows one to characterize activity from different neuronal populations in terms of cross-spectral density responses between the contacts of multi-unit probes occupying different layers:

(5) $$g_{Y}{\left(\omega \right)}_{i}=Y_{i}(\omega ,\theta ){Y_{j}}^{*}(\omega ,\theta )\;=\underset{k}{\sum }L(k,\varphi )T_{i}(k,\omega )g_{u}(k,\omega )T_{j}{\left(k,\omega \right)}^{†}L{\left(k,\varphi \right)}^{†}\;T_{i}(k,\omega )=\mathrm{FT}(K_{i}(\tau ))\;K_{i}(\tau )=\partial y_{i}(t,\theta )/\partial U(t-\tau )$$

where $g_{u}$ is the spectral density of endogenous neuronal input (parameterised as scale free noise as described in Pinotsis et al., 2014) and $T_{i}$ is the transfer function associated with the neural source; i.e., the Fourier Transform of impulse response function. This is known as the first order Volterra kernel $K_{i}$. Here, $Y_{i}(\omega ,\theta )=\mathrm{FT}(y_{i}(t,\theta ))$ is a Fourier transform of the equivalent time-series. Similarly to the neural mass model (4), compartmental models can describe activity from cortical columns consisting of excitatory and inhibitory populations. However, they consider this activity in more detail as generated by an ensemble of smaller structures called mini-columns. Below we will consider ten such mini-columns. In this setting, activity predicted from the compartmental model of (Jones et al., 2007) is a simple superposition of minicolumn activities

(6) $$\begin{array}{c} y_{i}(t,\theta )=\sum_{j\in \{q\}}y_{j}(t,\theta )\\ y_{j}(t,\theta )=y_{j}\left({\sum_{q'}A_{q'}l_{q'}L_{iq'}J_{q'}(t),Q\left(J_{k},L_{k},a_{kj},\vartheta \right)}_{\begin{array}{c} k\in \{q\}\\ k\neq q' \end{array}}\right) \end{array}$$

where the index q'⊆q runs over a subset of compartments *q* that defines each mini-column and j=1,...,10 runs over the mini-columns. In our case, each mini-column comprises the compartments of 1 superficial PN, 1 deep PN and superficial and deep interneurons, and $Q(J_{k},L_{k},a_{kj},\vartheta )$ stands for the exogenous input – that depends on activity in proximate compartments that is indexed by k∈{q},k≠q'. The argument in the factor *Q* in Equation 6.2 above simply means that this input depends upon the current density in the adjacent mini-columns, their lead fields, anatomical parameters ϑ and the strength of their connections $a_{kj}$.

**Fig. 2:**
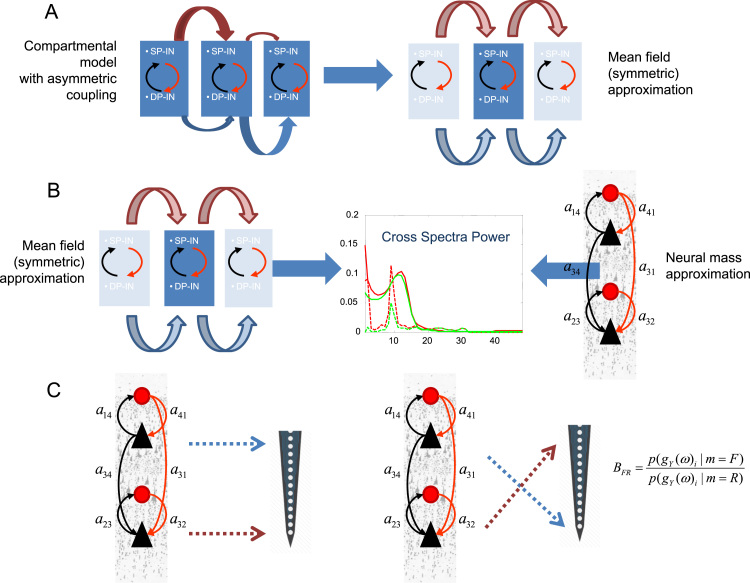
Schematic of the validation steps. A. We first establish the functional equivalence between the model of Jones et al. (2007) and its symmetric variant. Here horizontal arrows of different widths in the left panel denote asymmetric connectivities and delays between mini-columns depicted as rectangles containing Superficial and Deep Pyramidal cells (SP and DP) and Inhibitory Interneurons (II). In the right panel a symmetrisation of the model reveals a setup similar to one considered in mean field (neural mass) models B. We then demonstrate the construct validity of the corresponding mass model in relation to mean field model above. This is achieved by fitting the model to synthetic data obtained from its compartmental homologue. C. Finally, we show how this model can distinguish between superficial and deep responses obtained with laminar probes and consider the concomitant changes in model parameters with and without optogenetic manipulation. We exploit Bayesian model selection and compute the relative log-evidence for plausible (left) and implausible (right) experimental setups, where the probes of laminar sensors are considered in right and reversed locations, see the Results section.

Below, we assume that $a_{kj}$ are the same between any mini-column pair and that all mini-columns have the same structural characteristics. Intuitively, this could be thought as establishing an identity mapping between the activity of any pair of mini-columns *j* and *j’*, that defines an invariant subspace $y_{j}=y_{j'}$ in the full phase space of the network; see also Fig. 2. The rigorous proof of existence of such a subspace is a hard problem that goes beyond the scope of the current paper; see e.g. (Breakspear and Terry, 2002, Fujisaka and Yamada, 1983, Pecora and Carroll, 1990) for a discussion of this active area of research (Afraimovich et al., 2001, Breakspear et al., 2003).

In the first step of our analysis below, we first ensured that the symmetric version of the compartmental model produced the same responses as the original compartmental model used by (Jones et al., 2007). We then derived the homologous (neural mass) model by inverting the model in Fig. 1 to obtain (neural mass) parameters that best explain compartmental model responses in the Fourier domain. This exploits stationarity and ergodicity assumptions that allow us to quantify brain responses in terms of spectral densities. In the final step, we use these parameters as prior expectations for dynamic causal modelling of empirical data, using the neural mass or mean field model; see Fig. 2. This allowed us to establish the face validity of our model using responses recorded with laminar probes originating from different cortical layers; with and without experimental manipulation (optogenetics) – and investigate whether the model parameters show the expected experimental effects. A schematic summarising these steps is provided in Fig. 2.

The inversion of mean field models above uses the standard DCM approach, where inference on parameters and models is based on optimizing a free energy bound on the model log-evidence. Under Gaussian assumptions about the variational density q(θ)∼N(μ,C) and observation noise ε(μ)∼N(I,Σ(ω,λ)), the free energy has a very simple form:

(7) ℱ=G(μ)+12ln|∂μμG|G=−12Re(ε)T∑−1Re(ε)−12Im(ε)T∏Im(ε)−12ρTΩ−1ρ−12ln|∑|−12ln|Ω|ε=gY(ω,μ)+gN(ω,μ)−gY(ω)ρ=μ−ϕ

Here, $g_{Y}(\omega ,\mu )+g_{N}(\omega ,\mu )$ are the predictions of the data features $g_{Y}(\omega )$ and ρ(μ)∈ℝ are prediction errors on the parameters, in relation to their prior density p(θ|m)=N(ϕ,Ω) and Gis the Gibb's energy of the system.

The free energy bound is optimized with respect to a variational density q(θ) on the unknown model parameters. By construction, the free energy bound ensures that when the variational density maximizes free energy, it approximates the true posterior density over parameters, $q(\theta )\approx p(\theta |g_{Y}{\left(\omega \right)}_{i},m)$. At the same time, the free energy itself $ℱ(g_{Y}{\left(\omega \right)}_{i},q)\approx \;\ln\;p(g_{Y}{\left(\omega \right)}_{i}|m)$ approximates the log-evidence (marginal likelihood) of the data. In our final analysis below, we use the (relative) log-evidence to test whether our model can recover the sources of laminar recordings (originating from superficial and deep pyramidal cells). In Bayesian statistics, the relative log-evidence $B_{FR}$ plays a similar role to *p*-values in classical statistics. We computed $B_{FR}$ by fitting data to the neural mass model using: (i) the actual experimental setup (plausible spatial arrangement, m=F) and (ii) after reversing the mapping between superficial (deep) signals and deep (superficial) electrodes (implausible spatial arrangement, m=R). See also Fig. 2. The relative log-evidence is evaluated using the following expression (Kass and Raftery, 1995)

(8) $$B_{FR}=\;\ln\;\frac{p(g_{Y}{\left(\omega \right)}_{i}|m=F)}{p(g_{Y}{\left(\omega \right)}_{i}|m=R)}$$

where $p(g_{Y}{\left(\omega \right)}_{i}|m)$ is the evidence for the setup *m*. A relative log-evidence *B*_*FR*_>3 is taken as strong evidence for the forward (plausible) setup *F* over the implausible (reverse) setup.

## 3 Results

In the first part of analyses we repeated the analysis of (Jones et al., 2007) and modelled somatosensory evoked responses during a tactile stimulation paradigm. For these analyses we used two models: the model of (Jones et al., 2007) and a modified (simplified) version, which we call the symmetric model, see below. Our goal was to establish the equivalence between the original and simplified variants. This equivalence was established quantitatively by simulating responses of both models to the same input and ensuring that they generate the same evoked responses.

**Fig. 3:**
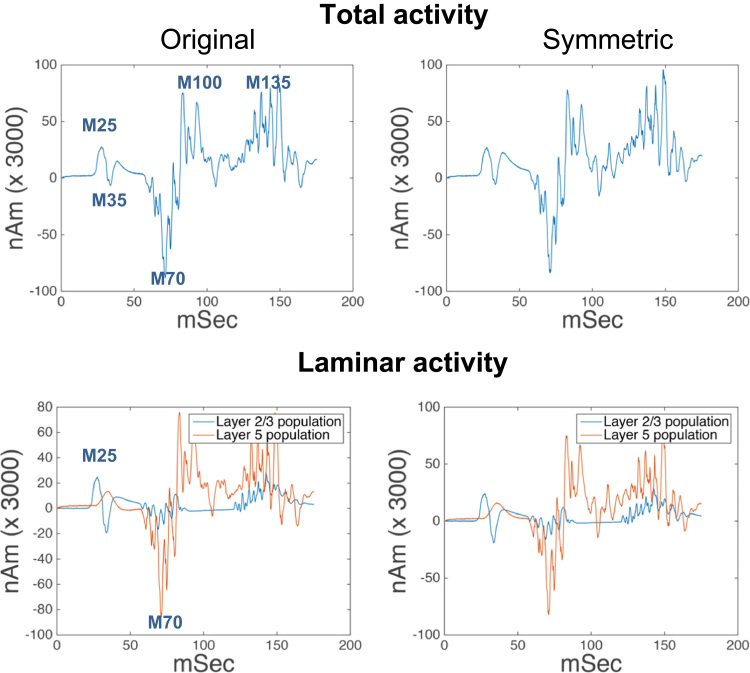
(Top) Simulated evoked responses of (left) the model used in Jones et al. (2007); (right) its symmetric variant. (Bottom) Contributions to the net dipole per layer for the same models; see Jones et al. (2007) for the corresponding results for the original model.

Both models were integrated 100 times and a simulated evoked response was obtained by averaging over trials as in (Jones et al., 2007). Fig. 3 (top row) shows the evoked response of the original model (left) and the symmetric model (right). The correlation coefficient between the two time series was r=0.9343, *p*<0.001, suggesting that the symmetric model was able to reproduce the evoked response to tactile stimulation as in (Jones et al., 2007). Crucially, the M25, M35, M50, M70, M100 and M135 peaks observed experimentally were all present in the simulated signal. Note that the response magnitude of the models has been multiplied by a scaling factor of 3000 to match the magnitude of the MEG response.

Fig. 3 (bottom row) shows the contributions to the net current dipole (CD) from each cortical layer. L5 PNs contribute more to the net CD than the L2 PNs, because of their longer apical dendrites. Furthermore, specific peaks in the net evoked response can be assigned to activity in specific cell types. For instance, the M25 peak seems to be primarily induced by activity in the L2 cells, while the large M70 peak can be attributed to activity in L5.

**Fig. 4:**
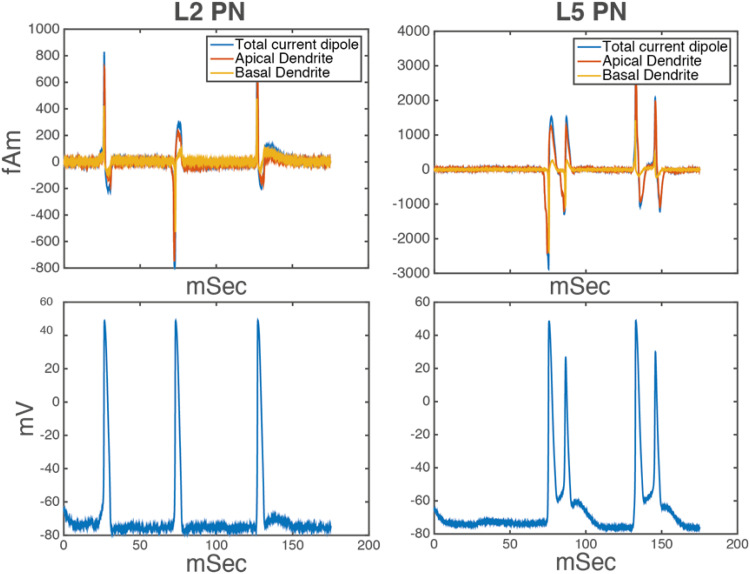
Activity of both PNs in the symmetric model. The top row shows the current dipoles of L2 and L5 neurons. The bottom row shows voltage responses; see Jones et al. (2007) for the corresponding results under the original (compartmental) model.

A more detailed picture emerges by studying the responses of specific dendritic compartments. Fig. 4 (top row) shows the contributions of apical and basal compartments to the CD of L2 and L5 PNs. The somatic potentials of both cells are plotted in the bottom row to illustrate the relationship between spikes and current flow. This is useful for characterising the origin of the peaks in the evoked response from the symmetric model and is very similar to the corresponding results that were obtained using the original model; see Jones et al. (2007).

The above analysis establishes the functional equivalence of the symmetric model (used below to simulate oscillatory responses) and the original Jones et al. model.

**Fig. 5:**
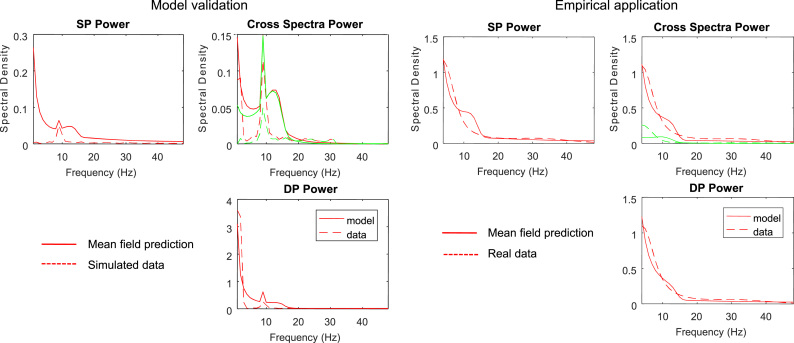
**Left panel:** Cross spectral density data from superficial and deep populations of the compartmental (Jones et al., 2007) model and model fits using its neural mass homologue. Model predictions are shown with solid lines and simulated data with dashed lines. Note the peaked responses at 10 Hz that are reminiscent of spiking burst input that are also captured by the mean field model responses. **Right panel:** Exemplar spectral responses and model fits obtained during the visual perception paradigm of (Pinto et al. 2013) from pairs of superficial and deep contacts across the thin laminar probe. These used bipolar data from V1 during optogenetic stimulation of the basal forebrain. Solid and dashed lines represent model fits and data. Red and green curves correspond to the real and imaginary parts of the cross spectral density respectively.

In the second part of our analyses, we asked whether the symmetric model can produce alpha activity. This investigation was motivated by the original (Jones et al., 2007) model which had been shown to produce alpha oscillations when a 10 Hz thalamic input was added to baseline noise (Jones et al., 2009). We reproduced the results of (Jones et al., 2009) using the symmetric model and obtained the simulated responses shown in the left panel of Fig. 5 (dashed lines). These responses were elicited by perturbing the local circuitry with ongoing rhythmic FF drive, where each burst consisted of 2 spikes with an inter-spike interval of 10 ms – and an inter-burst interval of (on average) 100 ms. We then focused on responses to thalamic input to the superficial and deep pyramidal cells. Fig. 5 shows the power spectra for both populations. These are the spectra generated by current flowing up and down the apical dendrites of the PNs.

Finally, we used the data from V1 during optogenetic stimulation of the basal forebrain (reported in the right panel of Fig. 5) to invert the neural mass model of Fig. 1 that is driven by endogenous noise. The resulting model fits are shown as solid lines in Fig. 5 (left panels). Interestingly, the 10 Hz peak evident in the simulated data is also captured in the predicted spectral responses. This reflects the fact that thalamic input to the neural mass model contains a specific 10 Hz peak. Following (Jones et al., 2009) we included a parameterised endogenous input to accommodate both the thalamic 10 Hz drive and baseline noise. In summary, we used responses generated by detailed dendritic morphologies to estimate the parameters of a mean field model, so that it could reproduce these spectral responses (cf. Table 3). We now consider the analysis of empirical data, using this mean field (neural mass) model.

### 3.1 Dynamic causal modelling of empirical data

In the final part of our analyses, we focused on real LFP data from (Pinto et al., 2013). These included power spectra obtained from the primary visual cortex during optogenetic stimulation of the basal forebrain. This allowed us to test two predictions from the DCM model of Fig. 1: whether it produces responses recorded from different cortical layers – and the increase in the excitability or gain of superficial pyramidal cells, due to cholinergic effects, as suggested by predictive coding.

A crucial (empirical) validation of the neural mass model rests on showing that the distinction between superficial and deep populations – based on their physiology and connectivity but not their spatial deployment – is valid in light of spatially resolved laminar data. Therefore, we compared a forward (F) model, in which superficial/deep populations are correctly assigned to supragranular/infragranular measurements, with a reverse (R) model, in which the assignment of modelled populations to their corresponding measurements has been switched.

Additionally, the optogenetic manipulation allowed us to address the face validity of the model using the natural (Laser OFF) and stimulated conditions (Laser ON); this allowed us to ask whether the model parameters change between conditions as we expected them to. In particular, cholinergic input to the cortex is known to disynaptically disinhibit layer 2/3 pyramidal cells through activation of layer 1 and vasoactive intestinal peptide-expressing inhibitory interneurons (Alitto and Dan, 2012, Fu et al., 2014, Lee et al., 2013, Letzkus et al., 2011, Pi et al., 2013). These are key tests for the validity of the neural mass model: whether it successfully distinguishes between the activities of superficial and deep pyramidal cells, and whether changes in connectivity model estimates capture the effects of cholinergic manipulation.

To address these questions, we inverted the neural mass model using the empirical LFP responses acquired from different depths (with and without optogenetic stimulation). For this analysis, we selected LFP channels 2 and 15 (second channels from the top and bottom of the array) from supragranular and infragranular layers respectively. The results of this inversion are shown in the right panels Fig. 5. To demonstrate that the model can successfully reproduce distinct deep vs. superficial activities, we used Bayesian model comparison. This allowed us to assess the quality of plausible and implausible spatial arrangements of the deep and superficial pyramidal cells and evaluate relative log-evidences as described above: equation (8).

Table 4 shows the results of Bayesian model comparison in terms of relative log-evidence, evaluated independently under both conditions (with and without optogenetic activation of the basal forebrain) when we swapped superficial and deep recordings around; i.e., fit the model with a plausible (forward) and implausible (reverse) laminar assignment of superficial and deep neuronal populations.

**Fig. 6:**
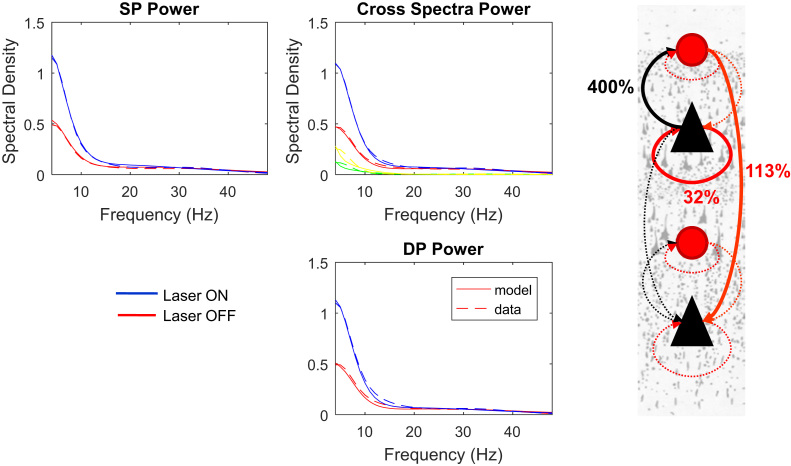
**Left panel:** Cross spectral density data responses and model fits obtained during the visual perception paradigm of (Pinto et al. 2013) from pairs of superficial and deep contacts across the thin laminar probe for Laser ON and Laser OFF conditions. This figure follows the format of the Right Panel of Fig. 5. Green and yellow curves correspond to imaginary parts of the cross spectral density for Laser OFF and Laser ON conditions respectively **Right panel:** Conditional parameter estimates and their trial specific changes: maximum a posteriori estimates of changes in coupling obtained after inverting data acquired with and without cholinergic stimulation are shown by the connections in question (using the same format as the insert in Fig. 1). Note the disinhibition of the superficial pyramidal cell population due to a decrease of inhibitory connectivity and the increase of the corresponding inhibitory influence on deep pyramidal cell populations. Of the ten intrinsic connections (see Fig. 1) we model condition specific changes in three (solid lines). The remaining connections were assumed to have the same values under both conditions, with prior expectations based upon the analysis of the compartmental model (dotted lines).

As noted above, a relative log-evidence (i.e., log Bayes factor) of three or more is taken as strong evidence for one model over another (Kass and Raftery, 1995). Bayesian model comparison suggests that the neural mass model distinguishes between responses originating from different layers, with substantially greater evidence for the plausible assignment of superficial pyramidal cells to supragranular layers. This was true with and without optogenetic manipulation. To ensure this result generalised over electrode pairs, we repeated the analysis for different deep and superficial channels and obtained the same result for every combination tested (results not reported). Finally, a quantitative comparison of the parameter estimates on and off stimulation suggested that cholinergic input enhances superficial relative to deep layer sensitivity (Fig. 6).

In the preceding analyses, we inverted both conditions separately (ON and OFF optogenetic stimulation) and compared models with a correct and incorrect laminar architecture. Our aim was to see if the model with the correct laminar disposition was selected by a Bayesian model comparison. In the final analysis, we illustrate the application of the neural mass model to answer questions about condition specific effects. In this instance, we model both conditions with the same (average) connectivity and test hypotheses about the condition specific effects by allowing them to operate on a subset of connections. This enables us to identify the precise changes in connectivity seen anecdotally in the condition specific versions.

In brief, the influence from superficial inhibitory populations on deep pyramidal cells increased by 113%, while the excitatory activity from superficial pyramidal cells increased by 400% during cholinergic stimulation. This was accompanied by a disinhibition of superficial pyramidal cells (decrease in self inhibition) by 32%. These self connections stand in for recurrent connections via inhibitory interneurons (e.g., parvalbumin positive Basket cells).

This is consistent with the emerging picture about the effects of cholinergic input and the experimentally observed facilitation of superficial-layer activity that could also be due to increased direct drive from layer 4 neurons (Douglas and Martin, 2004, Pluta et al., 2015), since feedforward thalamic input is enhanced by acetylcholine (Disney et al., 2007, Metherate and Ashe, 1993). These provisional results suggest that the segregation of ascending and descending streams of information might become less pronounced during optogenetic manipulation of cholinergic neurons, as a result of gain modulation of superficial principal cells through polysynaptic connections: in future work, we will investigate this hypothesis further using Bayesian model comparison: see also (Moran et al., 2013).

## 4 Discussion

We have introduced a neural mass model that can explain data obtained with thin laminar probes penetrating the cortex and sampling different cortical layers. We have tried to establish the construct validity of this neural mass model – in relation to more detailed compartmental models – by showing that the response profiles are formally equivalent in terms of evoked responses. We then addressed the face validity of the ensuing DCM by showing that it could identify the correct assignment of superficial pyramidal cells to supragranular layers and deep pyramidal cells to infragranular layers; irrespective of whether cholinergic (optogenetic) stimulation was present or absent.

We used data obtained during optogenetic activation of the basal forebrain in a visual perception paradigm to provide proof of principle that laminar specific recordings can be inverted using neural mass models – and that models of microscopic (invasive) data can inform hypotheses about interactions at a mesoscopic scale. A quantitative comparison of the parameter estimates suggests that cholinergic input enhances superficial activity, effectively boosting information ascending the cortical hierarchy. We hope to use the model introduced in this (technical) paper to pursue the (microscopic) functional anatomy of cholinergic modulation, using Bayesian model comparison in future work.

This model has been implemented as part of the DCM toolbox in the SPM freeware. The approach presented here can be used to address questions regarding laminar cortical microcircuitry that have so far remained inaccessible. In particular, we this model can be used to test (i) hypotheses about the function and structure of different neuronal populations at various depths of canonical microcircuits; (ii) functional architectures following from the predictive coding hypothesis. In the following, we consider these avenues for future research:

First, the neural mass model above may help us to better understand cortical anatomy and information processing: it enables one to test hypotheses about the function and structure of different neuronal populations in various cortical layers; e.g., evaluate differences in neural densities and cortical lamination (Balaram and Kaas, 2014, Slomianka et al., 2011). This can be achieved by considering differences in the connectivity parameters for the deep and superficial populations (parameters $a_{ij}$ in Table 3). By extending the model to a neural field similarly to (Pinotsis et al., 2012) one can also characterize the topography of connections in cortical hierarchies (Gattass et al., 2005).

Second, our model can be used to evaluate the evidence for – and test functional architectures following from the predictive coding hypothesis (Friston and Kiebel, 2009, Friston et al., 2015, Rao and Ballard, 1999). In particular, one can address open questions regarding a direct assignment of prediction error activity to a specific cortical layer. Also, in the particular context of optogenetic activation studies, as in the study by (Pinto et al., 2013) considered here, a detailed analysis of model parameters could also allow us to understand cortical circuit-level mechanisms of cholinergic modulation.

The key components of predictive coding – predictions, prediction errors, and precision – are often empirically studied in paradigms manipulating sensory expectation (Auksztulewicz and Friston, 2015) or attention (Feldman and Friston, 2010). Laminar characteristics of mismatch responses (Moran et al., 2013) and attentional effects (Auksztulewicz and Friston, 2015, Brown and Friston, 2012) have so far been inferred using DCM and non-invasively recorded data (FitzGerald et al., 2015). These studies have been supported by direct laminar recordings during attention tasks that yielded results consistent with the dissociation between superficial and deep layers; see e.g. (Buffalo et al., 2011). However, studies that exploit laminar recordings to consider different paradigms like mismatch responses are less conclusive (Fishman and Steinschneider, 2012, Natan et al., 2015), with similar evoked responses to sensory deviants in both supragranular and infragranular layers.

Using our model for the analysis of data from the sorts of studies referred to above, one can address questions about predictions, predictions errors and precision from a novel perspective: instead of analysing non-invasive or spatially unresolved data, the model can be used to exploit responses acquired at different cortical depths and study laminar-specific effects of cortical excitability that are crucial for understanding the balance between ascending and descending streams of information in the cortex (Bastos et al., 2012, Friston, 2010, Rao and Ballard, 1999). In summary, the new model presented may offer insights regarding the effects of expectation, attention and cholinergic neuromodulation.

Successfully addressing the questions described above rests upon validating models of the sort presented here. This rests upon technological advances in the construction of laminar probes and microelectrodes and developments in compartmental modelling. Modelling data recorded from penetrating microelectrodes promises a more direct window into the function of cortical microcircuits than that derived from recordings at the cortical surface (Bastos et al., 2015). Furthermore, building detailed compartmental and mean field models that capture important cortical computations and network biophysics is crucial for the success of the approach presented above. Starting with a new compartmental model one would then construct its mean field homologue and then validate it using the Bayesian procedure summarised in Fig. 2. This is the procedure we have applied for the case of the Jones et al. model.

More generally, a fuller understanding of cortical function is likely to depend upon successful characterization of the roles played by neurons in different cortical layers, and dynamic causal modelling may have the potential to further this aim. In future work, we will address these questions and test whether the laminar topography of current source density can be explained by interactions of superficial and deep PNs, by modelling spatially distributed lead fields, distinct spectral profiles and causal influences on network dynamics.

## Figures and Tables

**Table 1:** Synaptic connection parameters and strengths for the symmetric model.

	Max conductance (μS)	Weight space constant	Min. Delay (ms)	Delay space constant
L2/3 PN to L2/3 PN	0.001/0.0005	3	1	3
L2/3 PN to L2/3 IN	0.003	3	1	3
L2/3 PN to L5 PN	0.00025	3	3	3
L2/3 PN to L5 IN	0.000075	3	3	3
L2/3 IN to L2/3 PN	0.015/0.015	5	1	5
L2/3 IN to L5 PN	0.0003	5	1	5
L2/3 IN to L2/3 IN	0.0006	2	1	2
L5 PN to L5 PN	0.005/0.0005	3	1	3
L5 PN to L5 IN	0.0003	3	1	3
L5 IN to L5 PN	0.0075/0.0075	7	1	7
L5 IN to L5 IN	0.0006	2	1	2

**Table 2:** Suprathreshold synaptic weights for exogenous input.

	Maximal conductance (μS)
FF to L2/3 PN	0.002
FF to L2/3 IN	0.0012
FF to L5 PN	0.001
FF to L5 IN	0.0006
FB to L2/3 PN	0.004/0.004
FB to L2/3 IN	0.0006/0.0006
FB to L5 PN	0.004/0.004
LFF to L2/3 PN	0.08
LFF to L2/3 IN	0.024
LFF to L5 PN	0.04
LFF to L5 IN	0.012

**Table 3:** Prior expectations of parameters in the neural mass model of Fig. 1.

*Parameter*	*Physiological interpretation*	*Prior mean*
$\kappa_{1},\kappa_{2},\kappa_{3},\kappa_{4}$	Postsynaptic rate constants	1/2, 1/36, 1/16, 1/28 (ms^−^^1^)
$\alpha_{11},\alpha_{14},\alpha_{12}$		4,4,8
$\alpha_{22},\alpha_{21},\alpha_{23},\alpha_{33}$	Amplitude of intrinsic connectivity kernels	4,4,2,4 (a.u)
$\alpha_{41},\alpha_{32},\alpha_{44}$	(×200)	4,8,8
r,η	Parameters of the postsynaptic firing rate function	0.6, 0 (mV)

**Table 4:** *Log-evidence of neural models*. This table reports the relative Log-evidence for the forward (plausible) and reverse (implausible) model, using empirical data recorded during the Laser On (optogenetics stimulation) and Laser Off (no cholinergic stimulation) conditions.

Condition	Laser ON	Laser OFF
Forward Log-evidence		
$\log\;p(g_{Y}{\left(\omega \right)}_{i}|m=F)$	1498	2334
Reverse Log-evidence		
$\log\;p(g_{Y}{\left(\omega \right)}_{i}|m=R)$	1394	2201

## Footnotes

3 https://senselab.med.yale.edu/ModelDB/showmodel.cshtml?model=113732.
